# Dexmedetomidine vs. propofol for sedation in mechanically ventilated ICU patients: a systematic review and meta-analysis of randomized controlled trials

**DOI:** 10.3389/fneur.2026.1875240

**Published:** 2026-07-22

**Authors:** Xiao-Yan Zhang, Song Qin, Meng-Jun Liu, Yun-Yan Wang

**Affiliations:** 1 Department of ICU, Yunyang County People's Hospital, Chongqing, China; 2 Department of Orthopedics, Yunyang County People's Hospital, Chongqing, China

**Keywords:** bradycardia, delirium, dexmedetomidine, ICU sedation, mechanical ventilation, meta-analysis, propofol, randomized controlled trial

## Abstract

**Background:**

Dexmedetomidine and propofol are the two most widely used non-benzodiazepine sedatives for mechanically ventilated ICU patients. Despite guideline recommendations for both agents, the optimal choice remains controversial owing to inconsistent evidence across patient subpopulations.

**Methods:**

We conducted a systematic review and meta-analysis of randomized controlled trials (RCTs) directly comparing dexmedetomidine (DEX) with propofol (PROP) in adult ICU patients requiring mechanical ventilation. Databases searched included PubMed, Embase, Cochrane CENTRAL, and CNKI. Primary outcomes were duration of mechanical ventilation (MV) and ICU length of stay (LOS). Secondary outcomes included hospital LOS, all-cause mortality, delirium incidence, bradycardia, and hypotension. Random-effects models were applied using the restricted maximum likelihood (REML) estimator with Hartung–Knapp–Sidik–Jonkman (HKSJ) confidence interval correction. Risk of bias was evaluated using the Cochrane RoB 2.0 tool. Pre-specified subgroup analyses by ICU population type and sedation duration were performed.

**Results:**

Fifteen RCTs (*N* = 1,603; DEX: *n* = 815, PROP: *n* = 788; published 2001–2021) were included in the primary analysis. Four additional studies were included in sensitivity analyses. Dexmedetomidine did not significantly reduce MV duration (MD = −0.58 h; 95% CI: −2.10 to 0.95; *P* = 0.419; I^2^ = 67.1%) or ICU LOS (MD = −0.19 days; 95% CI: −0.67 to 0.29; *P* = 0.388; I^2^ = 63.3%). No significant difference in hospital LOS (MD = −0.21 days; *P* = 0.284; I^2^ = 0%) or all-cause mortality (OR = 0.92; 95% CI: 0.76–1.12; *P* = 0.354; I^2^ = 0%) was observed. Among secondary outcomes, dexmedetomidine was associated with lower delirium incidence (OR = 0.52; 95% CI: 0.33–0.80; *P* = 0.010; I^2^ = 27.6%). Bradycardia trended higher with dexmedetomidine (OR = 2.40; 95% CI: 0.94–6.17; *P* = 0.062) without reaching significance. Subgroup analysis revealed that ICU population type significantly moderated MV duration heterogeneity (interaction *P* = 0.004). Sedation duration also significantly moderated MV results (interaction *P* < 0.0001). The delirium reduction was directionally consistent across all population subgroups (interaction *P* = 0.508).

**Conclusion:**

In mechanically ventilated adult ICU patients, neither primary outcome (MV duration, ICU LOS) differed between dexmedetomidine and propofol. Dexmedetomidine may reduce ICU delirium incidence, but this secondary finding is borderline after correction for multiple comparisons and requires confirmation in dedicated trials. The higher bradycardia risk warrants vigilant haemodynamic monitoring during dexmedetomidine infusion.

**Systematic review registration:**

https://www.crd.york.ac.uk/PROSPERO/view/CRD420261378906, identifier: CRD420261378906.

## Highlights

- Dexmedetomidine and propofol are both recommended first-line non-benzodiazepine sedatives for mechanically ventilated adult ICU patients; however, direct head-to-head evidence comparing their effects on clinical outcomes remains heterogeneous across ICU subpopulations.
- This meta-analysis of 15 randomized controlled trials (*N* = 1,603 patients) found no statistically significant differences in the two pre-specified primary outcomes (MV duration and ICU LOS). Dexmedetomidine may reduce ICU delirium risk compared with propofol (OR = 0.52; 95% CI: 0.33–0.80; *P* = 0.010; I^2^ = 27.6%), with no statistically significant differences in mechanical ventilation duration, ICU or hospital length of stay, or all-cause mortality.
- Dexmedetomidine is associated with numerically higher rates of bradycardia (OR = 2.40) and hypotension (OR = 1.64), though neither reached statistical significance, likely due to insufficient event counts; the delirium benefit appears consistent across both cardiac surgical and mixed ICU subgroups.

## 1 Introduction

Sedation is a cornerstone of intensive care for mechanically ventilated patients, facilitating ventilator synchrony, reducing distress, and enabling essential clinical procedures. Achieving an appropriate sedation depth is, however, inherently difficult: under-sedation leads to patient–ventilator dyssynchrony, self-extubation, and physiological stress responses, while over-sedation independently prolongs mechanical ventilation (MV), increases ICU length of stay (LOS), and contributes to ICU-acquired delirium, long-term cognitive impairment, and mortality (1, 2).

Propofol (PROP), a γ-aminobutyric acid (GABA)-mediated sedative-hypnotic, has been the standard ICU sedative for decades, valued for its rapid onset, predictable titratability, and short context-sensitive half-life (1, 3). However, its use is associated with propofol infusion syndrome at high doses or prolonged infusion, as well as haemodynamic instability and hypertriglyceridaemia (2). Dexmedetomidine (DEX), a selective α2-adrenergic receptor agonist, has emerged as an important alternative offering a qualitatively distinct form of sedation: it preserves respiratory drive, allows arousable and cooperative sedation, and provides mild analgesia and sympatholysis through a non-GABA mechanism, which may underlie its purported advantage in preventing delirium (4, 5).

The 2018 SCCM PADIS guidelines recommend non-benzodiazepine sedation (propofol or dexmedetomidine) as first-line therapy for mechanically ventilated ICU patients and conditionally suggest dexmedetomidine to reduce delirium (6). Nevertheless, clinical equipoise persists between the two agents, as guideline recommendations do not specify which is preferable when both are available. Systematic reviews comparing DEX with PROP have produced inconsistent results, partly because many included trials with non-propofol comparators (e.g., midazolam, morphine, or placebo) (5, 7, 8), pooled heterogeneous ICU patient types without stratification, or used statistical methods susceptible to false-positive findings with small numbers of studies.

Since these earlier reviews, several landmark RCTs have been completed: the PRODEX trial (4), the MENDS2 trial (9), and the SPICE III trial (10), substantially expanding the available evidence base. The present systematic review and meta-analysis was therefore conducted to synthesize updated evidence exclusively from head-to-head DEX vs. PROP RCTs in adult mechanically ventilated ICU patients, applying REML estimation and HKSJ confidence interval correction, with pre-specified subgroup analyses by patient population type and sedation duration to characterize heterogeneity sources.

## 2 Methods

### 2.1 Literature search and eligibility criteria

**Figure 1:**
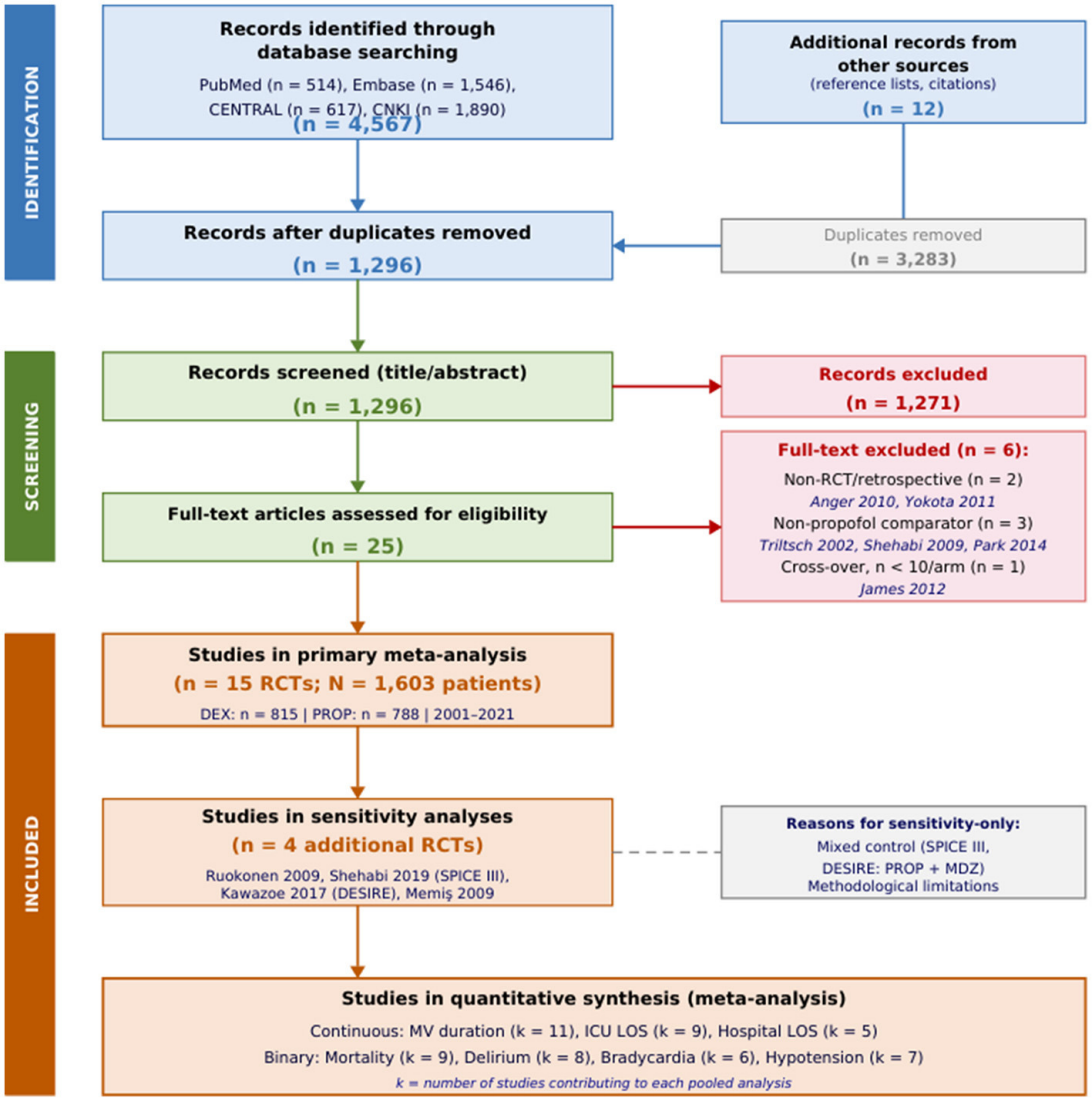
PRISMA 2020 flow diagram of study selection. Records were identified through PubMed, Embase, Cochrane CENTRAL, and CNKI. Fifteen RCTs (*N* = 1,603) were included in the primary meta-analysis and four additional studies in sensitivity analyses.

This review was conducted in accordance with the PRISMA 2020 guidelines and was prospectively registered in PROSPERO (CRD420261378906). Systematic electronic searches were performed in PubMed, Embase, Cochrane Central Register of Controlled Trials (CENTRAL), and CNKI up to the date of data extraction. Search terms included: dexmedetomidine, propofol, ICU sedation, mechanical ventilation, critically ill, and related MeSH/Emtree terms. Studies were eligible if they: (i) were RCTs; (ii) enrolled adult ICU patients (≥18 years) receiving invasive mechanical ventilation; (iii) directly compared dexmedetomidine with propofol as the primary sedative agent (head-to-head); and (iv) reported at least one pre-specified outcome. Exclusion criteria were: non-propofol comparator (e.g., midazolam, morphine, placebo) (5, 7, 8, 11); cross-over design with ≤ 10 patients per arm (12); non-randomized or retrospective design (13); and absence of extractable data. No language restrictions were applied. Excluded studies with reasons are presented in the PRISMA flow diagram (Figure 1).

### 2.2 Data extraction

Two reviewers independently extracted data using pre-specified forms. Extracted items included: study identification (author, year, country, design, multicentre status, sample size), patient characteristics (age, ICU population type, sedation target score), intervention details (DEX and PROP dosing, sedation duration category), and outcome data. For continuous outcomes (MV duration, ICU LOS, hospital LOS), means and standard deviations (SD) were extracted. Where outcomes were reported as medians with interquartile ranges, conversion to mean ± SD was performed using the formula of Wan et al. (14). This method assumes approximate normality and may bias estimates for strongly right-skewed distributions. For MV duration, all data were harmonized to hours; studies reporting in days were converted by multiplying the mean and SD by 24. For ICU LOS, all data were harmonized to days after exclusion of Elgebaly 2018. For binary outcomes (mortality, delirium, bradycardia, hypotension), event counts and denominators were extracted. Disagreements were resolved by consensus. A study-by-outcome inclusion matrix is provided as Supplementary Table S1.

### 2.3 Risk of bias assessment

**Figure 2:**
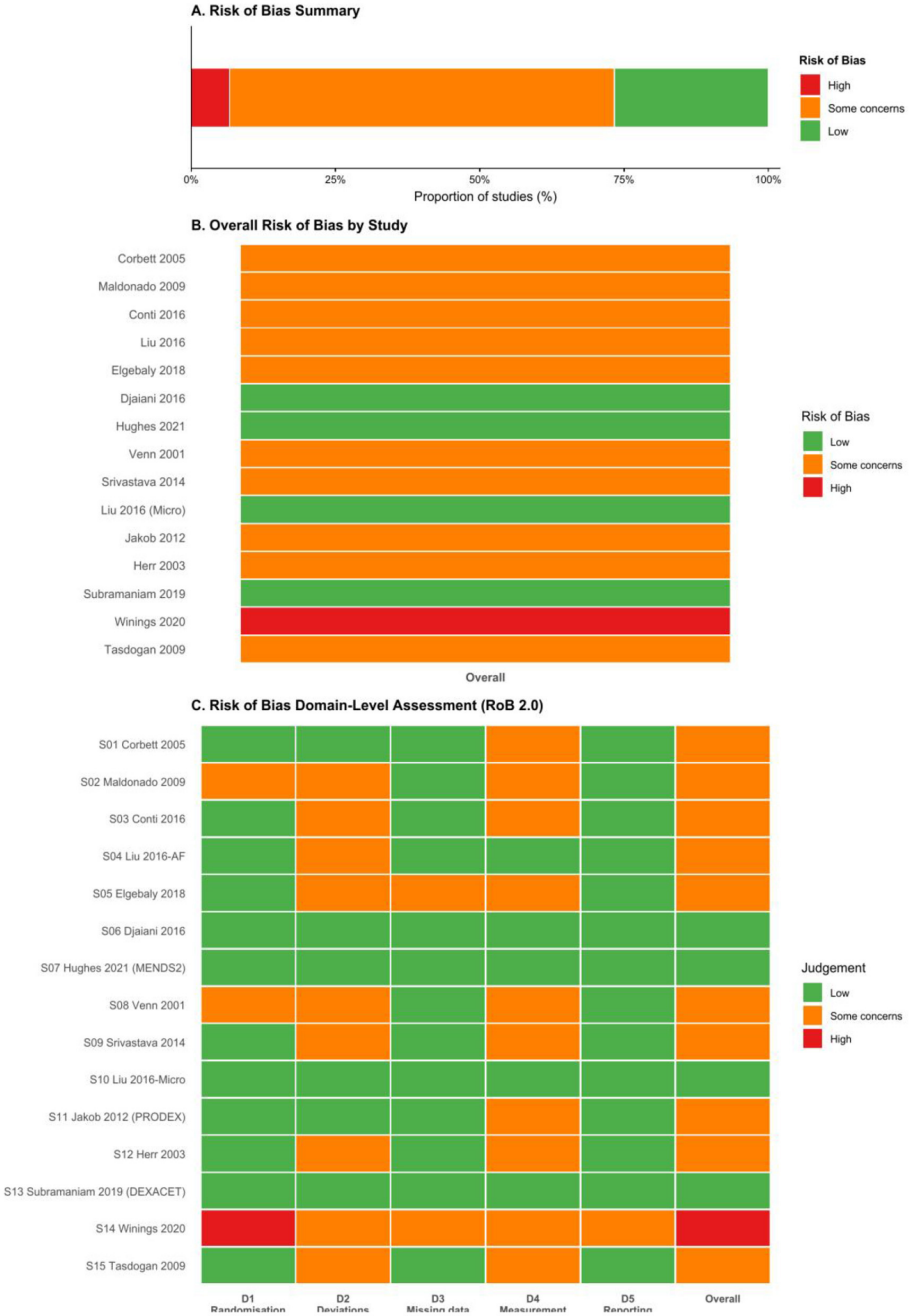
Risk of bias assessment (Cochrane RoB 2.0). **(A)** Stacked bar chart of the overall risk-of-bias distribution across the 15 primary-analysis studies, using the three canonical RoB 2.0 categories (low risk, some concerns, high risk). **(B)** Per-study overall risk-of-bias judgement. **(C)** Domain-level assessment of all five RoB 2.0 domains (D1, randomization; D2, deviations from intended interventions; D3, missing outcome data; D4, measurement of the outcome; D5, selection of the reported result) for each study.

Methodological quality was assessed independently and in duplicate using the revised Cochrane Risk of Bias tool (RoB 2.0), covering five domains: (1). randomization process; (2). deviations from intended interventions; (3). missing outcome data; (4). measurement of the outcome; and 5. selection of the reported result. Each study was classified as low risk, some concerns, or high risk of bias. Summary results are presented as a bar chart and a per-study heatmap (Figure 2).

### 2.4 Statistical analysis

#### 2.4.1 Effect size selection

Pooled mean differences (MD) with 95% confidence intervals (CI) were used for continuous outcomes; pooled odds ratios (OR) with 95% CI for binary outcomes. All analyses used random-effects models. The between-study variance (τ^2^) was estimated using the restricted maximum likelihood (REML) method, and confidence intervals were corrected using the Hartung–Knapp–Sidik–Jonkman (HKSJ) adjustment to improve coverage accuracy, particularly when the number of contributing studies is small.

#### 2.4.2 Heterogeneity assessment

Between-study heterogeneity was quantified using Cochran's Q test and the I^2^ statistic. I^2^ < 25%, 25–50%, and >50% were interpreted as low, moderate, and substantial heterogeneity, respectively. When substantial heterogeneity was identified, potential sources were explored through subgroup analyses and meta-regression.

#### 2.4.3 Subgroup analyses

Pre-specified subgroup analyses were conducted for: (i) patient population type (cardiac surgical, mixed ICU, sepsis, surgical), examining MV duration and delirium incidence; and (ii) sedation duration category (short < 24 h, intermediate 24–72 h, long >72 h), examining MV duration. Between-subgroup heterogeneity was assessed using the χ^2^ interaction test.

#### 2.4.4 Sensitivity analyses

A pre-planned sensitivity analysis incorporated four additional studies excluded from the primary analysis owing to mixed comparator designs or methodological limitations: Ruokonen and colleagues (15), Shehabi and colleagues [(10); SPICE III], Kawazoe and colleagues [(16); DESIRE], and Memiş and colleagues (17). MV duration and all-cause mortality were re-pooled.

#### 2.4.5 Publication bias

Funnel plots were constructed for all outcomes with ≥5 contributing studies. Egger's linear regression test was applied where ≥10 studies were available. All analyses were performed in R (version 4.4.2) using the meta (v8.3-0) and metafor (v4.8-0) packages. Statistical significance was defined as two-sided *P* < 0.05.

## 3 Results

### 3.1 Characteristics of included studies

The primary meta-analysis included 15 RCTs comprising 1,603 patients (DEX: *n* = 815; PROP: *n* = 788), published between 2001 and 2021 across the USA, Europe, China, the Middle East, and other countries (1, 3, 4, 9, 18–28). Six studies were multicentre. ICU populations comprised: post-cardiac surgical (7 studies), mixed ICU (2 studies), sepsis ICU (2 studies), and surgical/neurosurgical ICU (2 studies). Sedation duration was short (< 24 h) in 9 studies, intermediate (24–72 h) in 2, and long (>72 h) in 2. Risk of bias assessment: 4 studies rated low risk, 10 some concerns, 1 high risk (18). Full study characteristics are presented in Table 1. Four studies were reserved for sensitivity analyses: Ruokonen (15), Shehabi (10) (SPICE III), Kawazoe (16) (DESIRE), and Memiş (17). Six studies were excluded from the primary analysis: Triltsch (7) (placebo comparator), Shehabi (8)/DEXCOM (morphine comparator), Anger (13) (retrospective), Park (29) (non-propofol comparator), Yokota (30) (retrospective observational), and James (12) (crossover, *n* < 10 per arm).

**Table 1:** Characteristics of the 15 studies included in the primary meta-analysis.

Study	Country	Design	Multi-ctr.	*N*	*n* (DEX)	*n* (PROP)	Population	Age DEX (yr)	Age PROP (yr)	Sedation duration	Risk of bias
Corbett et al. (1)	USA	DB RCT	No	89	43	46	Post-CABG cardiac ICU	63.6 ± 10.1	62.4 ± 10.7	Short (<24 h)	Some concerns
Maldonado et al. (19)	USA	OL RCT	No	60	30	30	Cardiac valve surgery + CPB	55.0 ± 16.0	58.0 ± 18.0	Intermediate (24–72 h)	Some concerns
Conti et al. (22)	Italy	OL RCT	Yes	20	10	10	Mixed ICU; difficult weaning	68.8 ± 15.7	68.8 ± 15.7	Intermediate (24–72 h)	Some concerns
Liu et al. 2016-AF (23)	China	OL RCT	No	88	44	44	Post-cardiac surgery + CPB	53.0 ± 8.0	56.5 ± 8.0	Short (<24 h)	Some concerns
Elgebaly and Sabry (24)	Egypt	OL RCT	No	50	25	25	Post-cardiac/vascular surgery ICU	53.7 ± 6.1	52.5 ± 7.4	Short (<24 h)	Some concerns
Djaiani et al. (25)	Canada	SB RCT	No	183	91	92	Elderly (≥60yr) cardiac surgery	72.7 ± 6.4	72.4 ± 6.2	Short (<24 h)	Low
Hughes et al. (9)	USA (13 ctrs)	DB RCT	Yes	422	214	208	Sepsis ICU (MENDS2)	59.0 ± 10.0	60.0 ± 9.0	Long (>72 h)	Low
Venn and Grounds (27)	UK	OL RCT	No	20	10	10	Post-abdominal/pelvic surgery ICU	65.0 ± 9.0	67.0 ± 7.0	Short (<24 h)	Some concerns
Srivastava et al. (26)	India	SB RCT	No	57	28	29	Post-neurosurgery ICU	50.5 ± 7.4	52.1 ± 8.5	Short (<24 h)	Some concerns
Liu et al. 2016-Micro (28)	China	SB RCT	No	61	29	32	Cardiac valve surgery + CPB	53.0 ± 8.0	55.0 ± 8.0	Short (<24 h)	Low
Jakob et al. (4)	Europe + Russia (31 ctrs)	DB RCT	Yes	498	251	247	Mixed ICU; MV ≥24h (PRODEX)	65.0 ± 12.0	65.0 ± 12.0	Long (>72 h)	Some concerns
Herr et al. (3)	USA/Canada (25 ctrs)	OL RCT	Yes	295	148	147	Post-CABG cardiac ICU	61.9 ± 9.5	62.4 ± 8.7	Short (<24 h)	Some concerns
Subramaniam et al. (20)	USA	DB RCT	No	120	59	61	Elderly cardiac surgery (DEXACET)	66.0 ± 7.0	71.0 ± 8.0	Short (<24 h)	Low
Winings et al. (18)	USA	OL RCT	No	57	28	29	Trauma/surgical ICU	53.2 ± 18.1	48.2 ± 20.2	Long (>72 h)	High
Tasdogan et al. (21)	Turkey	OL RCT	No	40	20	20	Severe sepsis post-abdominal surgery	58.0 ± 15.0	50.0 ± 16.0	Short (<24 h)	Some concerns

DB, double-blind; OL, open-label; SB, single-blind; CPB, cardiopulmonary bypass; ICU, intensive care unit; DEX, dexmedetomidine; PROP, propofol; RoB, Cochrane RoB 2.0. Sedation duration: short < 24 h; intermediate 24–72 h; long >72 h. Reference numbers shown in brackets.

### 3.2 Continuous outcomes

#### 3.2.1 Duration of mechanical ventilation

**Figure 3:**
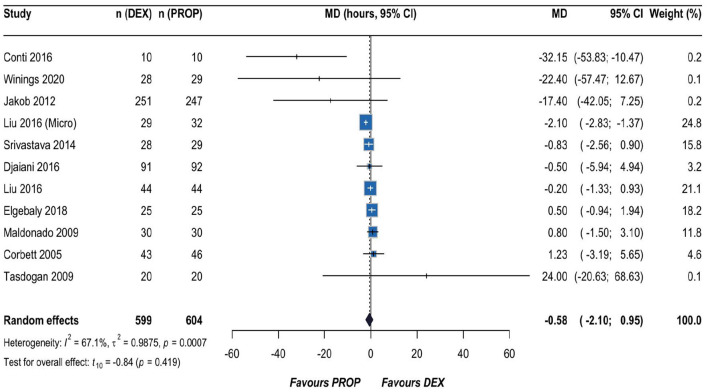
Duration of mechanical ventilation — forest plot (dexmedetomidine vs. propofol). All data in hours. MD, mean difference; CI, confidence interval. Random-effects model, REML + HKSJ adjustment. Negative MD favors dexmedetomidine.

Eleven studies (1, 4, 18, 19, 21–26, 28) (DEX: *n* = 599; PROP: *n* = 604) reported MV duration data. Under the random-effects model, there was no statistically significant difference between dexmedetomidine and propofol in MV duration (MD = −0.58 h; 95% CI: −2.10 to 0.95; *P* = 0.419). Substantial between-study heterogeneity was present (I^2^ = 67.1%; τ^2^ = 0.99; *P* = 0.0007), indicating important differences among studies in terms of patient population, sedation protocols, and outcome measurement approaches. The caution required in interpreting the pooled estimate is therefore considerable. At the level of individual studies, Conti 2016 (22) (MD = −32.15 h) represented a substantial outlier, driven by its mixed ICU, difficult-weaning population, which differed markedly from the predominantly cardiac surgical studies. In contrast, cardiac surgical studies [Liu 2016-Micro (28), Liu 2016-AF (23)] produced effect sizes clustered near the null. The forest plot is presented in Figure 3.

#### 3.2.2 ICU length of stay

**Figure 4:**
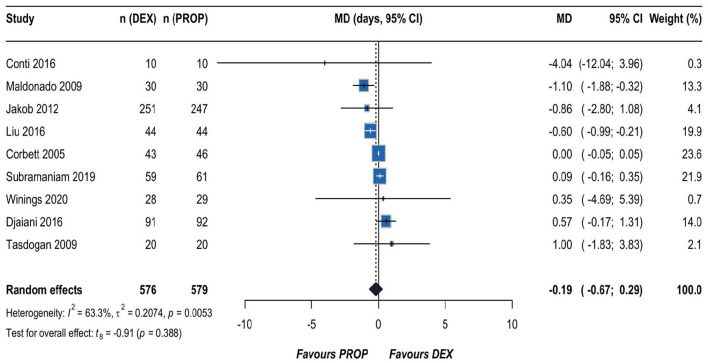
ICU length of stay — forest plot (dexmedetomidine vs. propofol). All data in days [Elgebaly (24) excluded]. MD, mean difference; CI, confidence interval.

Nine studies (1, 4, 18–23, 25) (DEX: *n* = 576; PROP: *n* = 579) reported ICU LOS after exclusion of Elgebaly (24), whose brief observation window was qualitatively dissimilar from multi-day ICU stays. All data were harmonized to days. No significant between-group difference was identified (MD = −0.19 days; 95% CI: −0.67 to 0.29; *P* = 0.388). Moderate-to-high heterogeneity was present (I^2^ = 63.3%; *P* = 0.0053). Residual heterogeneity was driven chiefly by differences in surgical population and post-operative care pathways across the remaining trials. A sensitivity analysis that re-included Elgebaly 2018 is provided in the Supplementary Material (Supplementary Figure S3); in that analysis Elgebaly contributed 76.8% of the weight and the pooled estimate was essentially unchanged (MD = −0.02 days; 95% CI: −0.05 to 0.02), confirming that its exclusion does not materially alter the conclusion. The forest plot for the primary analysis is presented in Figure 4.

#### 3.2.3 Hospital length of stay

**Figure 5:**
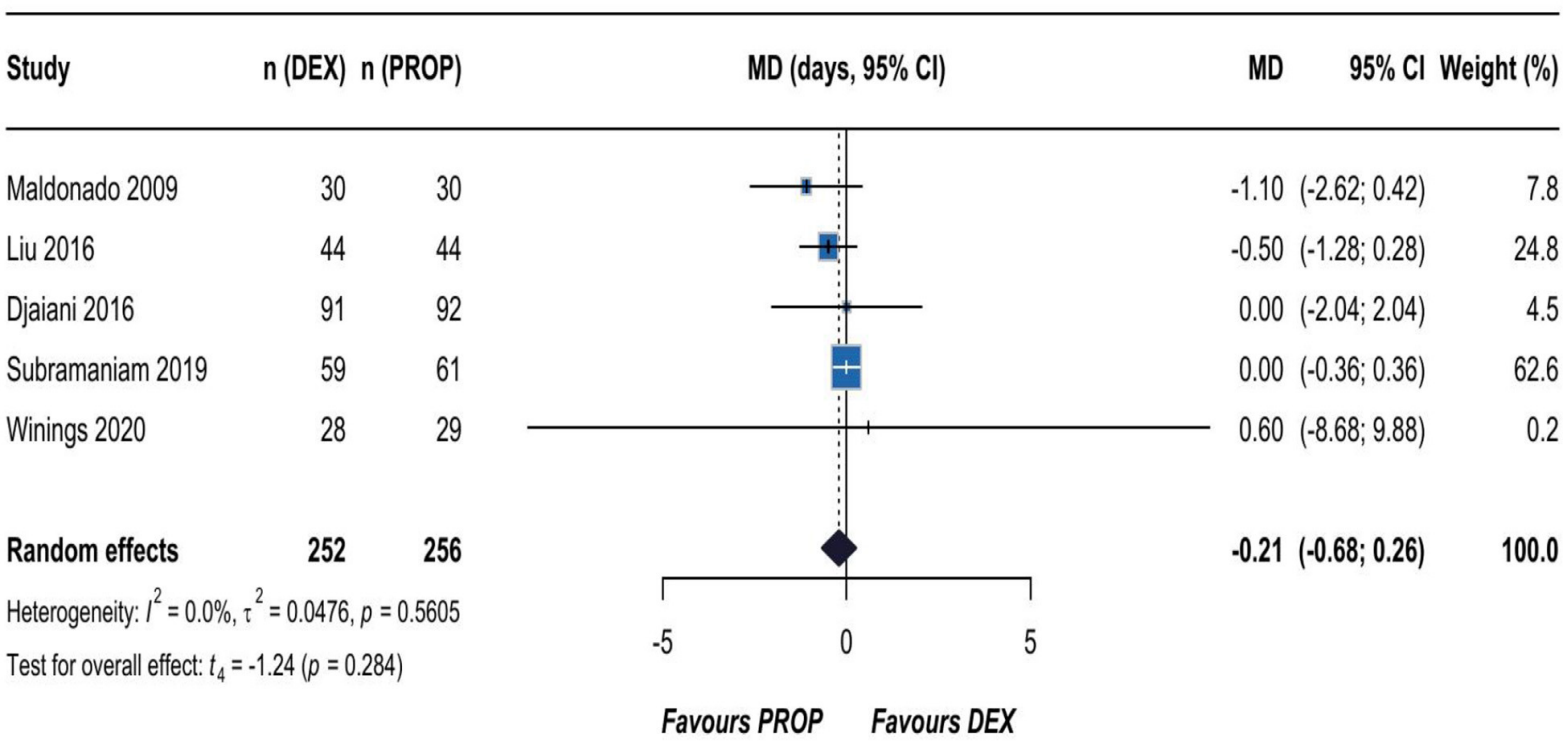
Hospital length of stay — forest plot (dexmedetomidine vs. propofol). All data in days. MD, mean difference; CI, confidence interval.

Five studies (18–20, 23, 25) (DEX: *n* = 252; PROP: *n* = 256) reported hospital LOS. There was no statistically significant difference (MD = −0.21 days; 95% CI: −0.68 to 0.26; *P* = 0.284; I^2^ = 0%). The low heterogeneity and consistent effect direction across studies support the robustness of this null finding. Subramaniam (20) (DEXACET) contributed 62.6% of the pooled weight; both arms had a median hospital LOS of 8 days with a difference of zero. The forest plot is presented in Figure 5. Pooled estimates for all three continuous outcomes are summarized in Table 2.

**Table 2:** Summary of meta-analysis results for continuous outcomes.

Outcome	Unit	Studies (k)	*N*	MD (95% CI)	*P*	I^2^ (%)	*P* heterogeneity
MV duration	Hours	11	1,203	−0.58 (−2.10, 0.95)	0.419	67.1%	<0.001
ICU length of stay	Days	9	1,155	−0.19 (−0.67, 0.29)	0.388	63.3%	0.005
Hospital length of stay	Days	5	508	−0.21 (−0.68, 0.26)	0.284	0.0%	0.560

MD, mean difference; CI, confidence interval; I^2^, inconsistency statistic; τ^2^, between-study variance. REML estimator with HKSJ confidence interval adjustment. Negative MD favors dexmedetomidine.

### 3.3 Binary outcomes

#### 3.3.1 All-cause mortality

**Figure 6:**
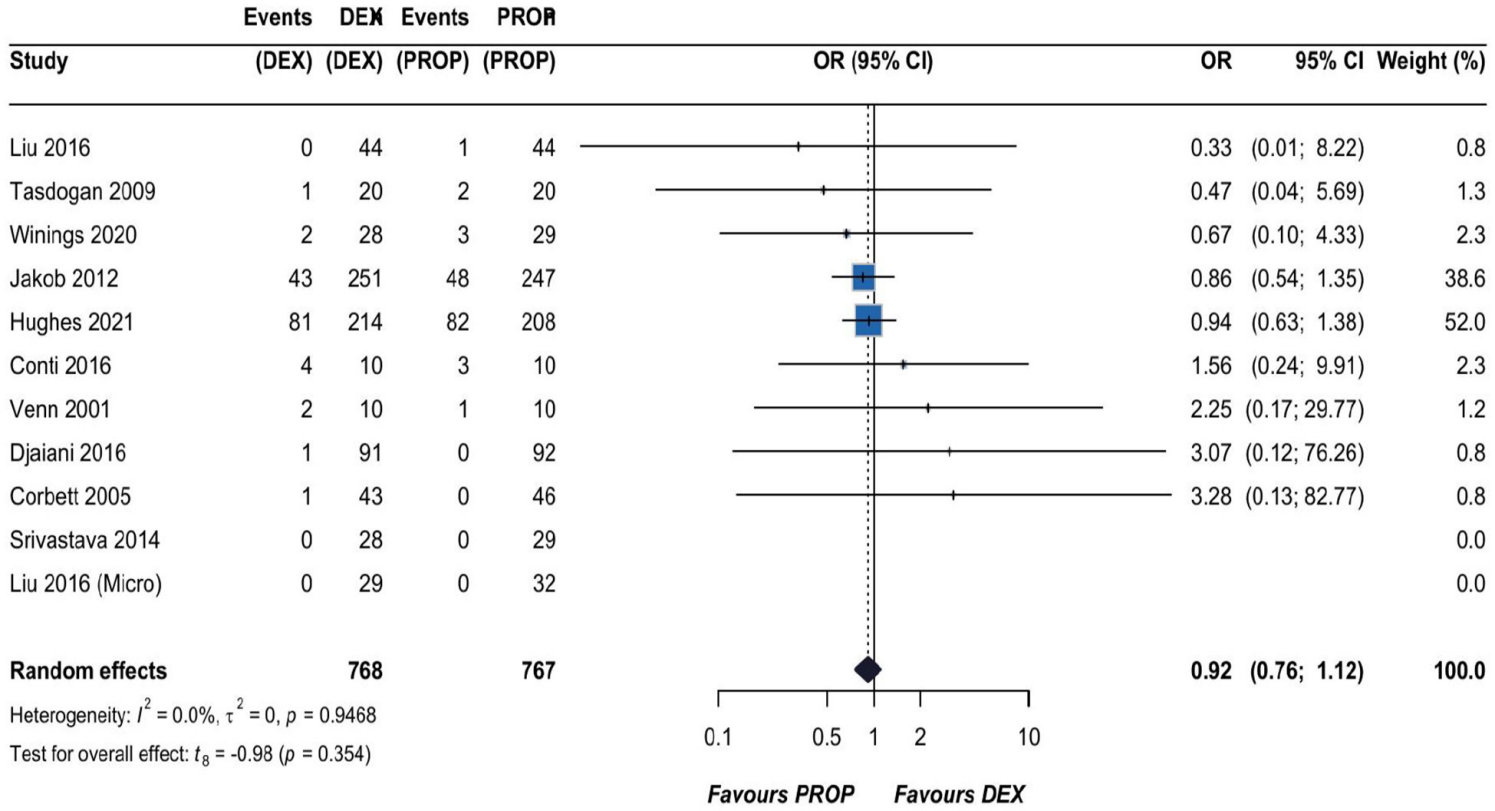
All-cause mortality — forest plot (dexmedetomidine vs propofol). OR, odds ratio; CI, confidence interval. OR < 1 favors dexmedetomidine.

Eleven studies reported mortality data;nine were included in the pooled analysis (1, 4, 9, 18, 21–23, 25, 27) (DEX: *n* = 768; PROP: *n* = 767), after excluding two studies with zero events in both arms [Srivastava (26) and Liu 2016-Micro (28)]. There was no statistically significant difference in all-cause mortality between groups (OR = 0.92; 95% CI: 0.76–1.12; *P* = 0.354). Between-study heterogeneity was negligible (I^2^ = 0%; Phet = 0.947), indicating a high degree of consistency across studies. The two highest-weighted studies—Hughes (9) (MENDS2), weight 52.0%, OR = 0.94, and Jakob (4) (PRODEX), weight 38.6%, OR = 0.86—both directionally favored dexmedetomidine but with confidence intervals crossing the null. The forest plot is presented in Figure 6.

#### 3.3.2 Delirium incidence

Eight studies (1, 4, 18–20, 23, 25, 28) (DEX: *n* = 575; PROP: *n* = 581) reported the incidence of ICU delirium assessed using validated instruments (CAM-ICU, CAM, or DSM-IV criteria).

**Figure 7:**
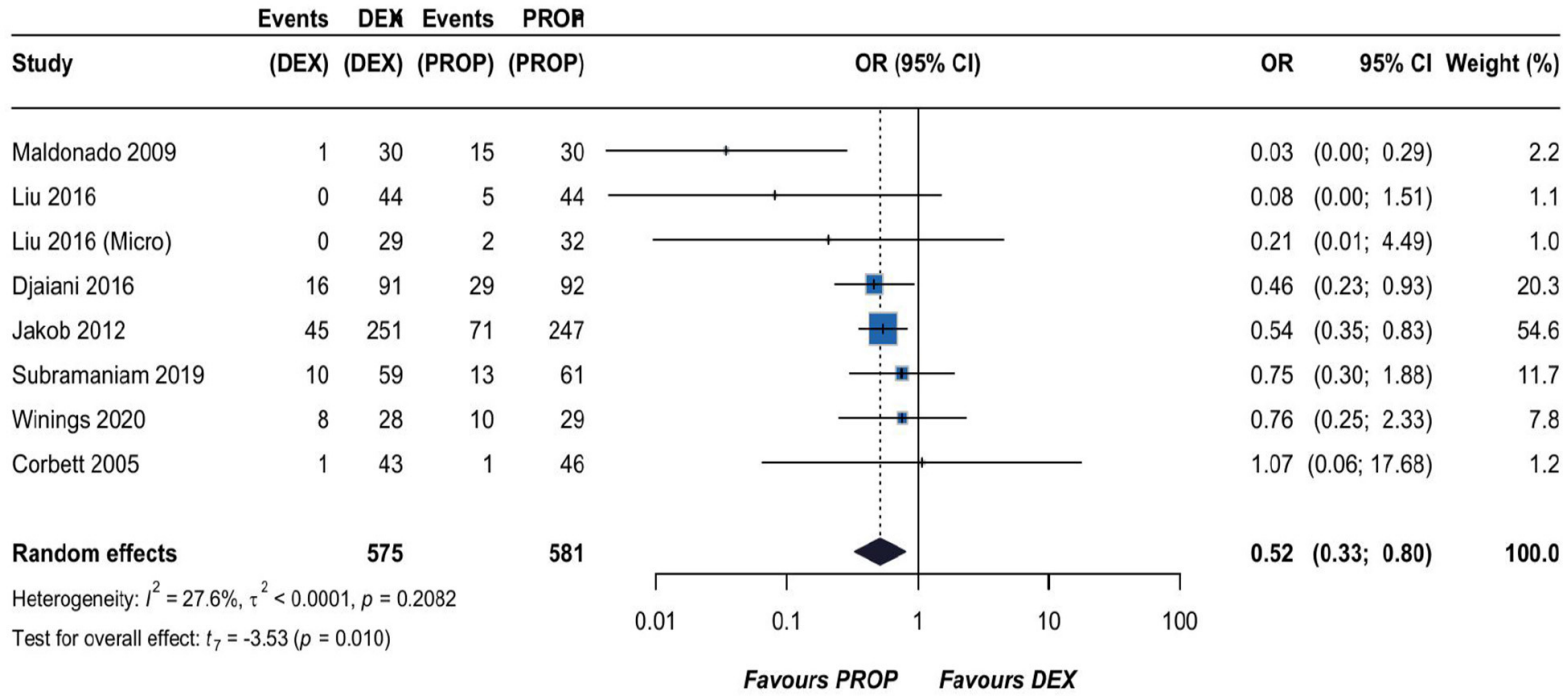
Delirium incidence — forest plot (dexmedetomidine vs. propofol). OR, odds ratio; CI, confidence interval. OR < 1 favors dexmedetomidine. Delirium assessed using CAM-ICU, CAM, or DSM-IV.

Dexmedetomidine was associated with a significantly lower risk of ICU delirium compared with propofol (OR = 0.52; 95% CI: 0.33–0.80; *P* = 0.010), corresponding to approximately 48% lower odds of delirium. Between-study heterogeneity was low to moderate (I^2^ = 27.6%; *P* = 0.208), indicating reasonable consistency of findings. The two highest-weighted studies—Jakob (4) (PRODEX) (weight 54.6%; OR = 0.54) and Djaiani (25) (weight 20.3%; OR = 0.46)—both demonstrated statistically significant reductions in delirium with dexmedetomidine, with consistent effect directions. This secondary outcome achieved the lowest *P*-value among the seven tested outcomes; however, with seven comparisons, a Bonferroni-adjusted threshold would be approximately 0.007, which this result does not meet. The delirium finding should be regarded as a hypothesis-supporting signal consistent with prior literature rather than a definitive conclusion. The forest plot is presented in Figure 7.

#### 3.3.3 Bradycardia

**Figure 8:**
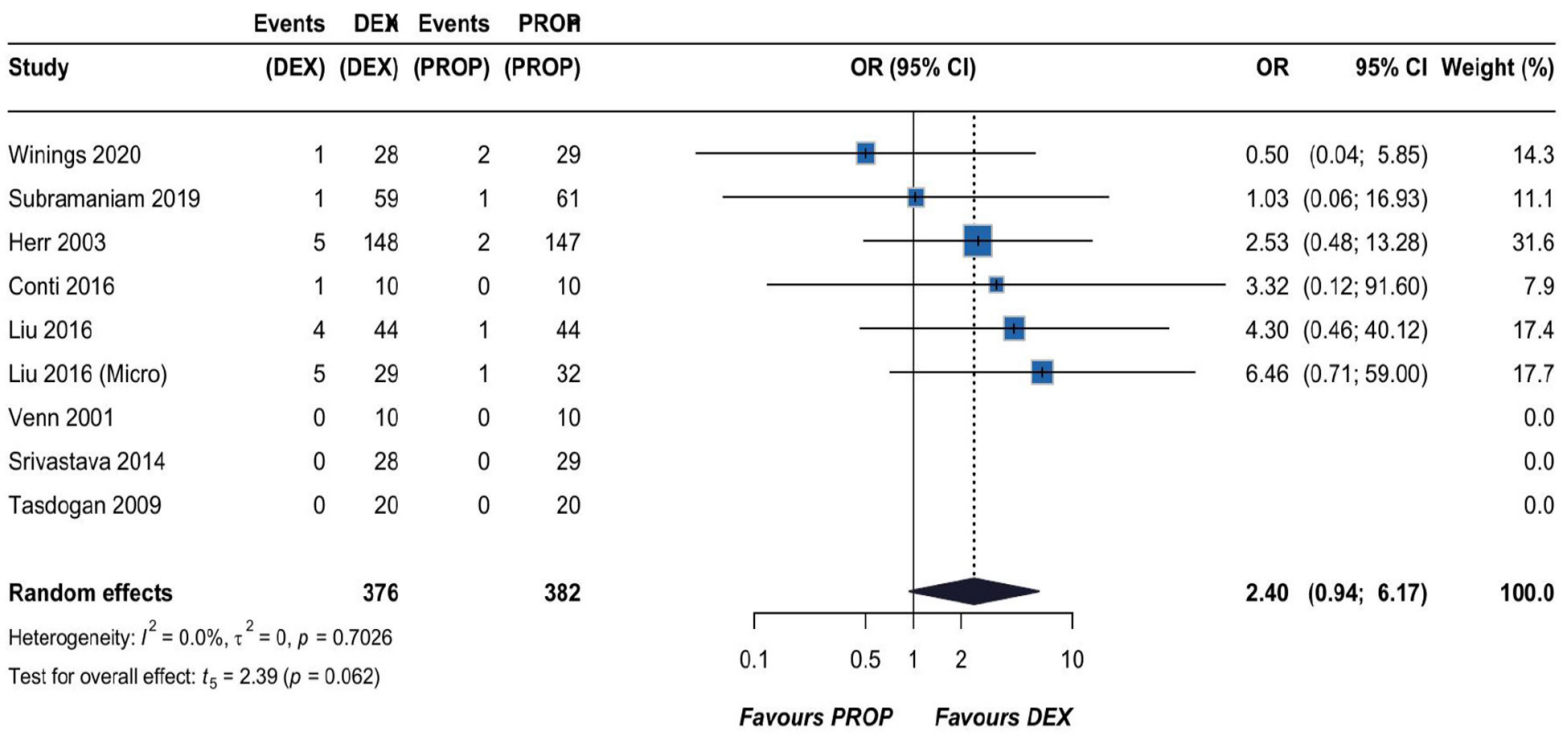
Bradycardia — forest plot (dexmedetomidine vs. propofol). OR, odds ratio; CI, confidence interval. OR > 1 favors propofol (lower risk).

Nine studies had bradycardia data; six (3, 18, 20, 22, 23, 28) (DEX: *n* = 376; PROP: *n* = 382) were included in the pooled analysis after excluding three studies with zero events in both arms. The pooled estimate showed a non-statistically significant trend toward higher bradycardia risk with dexmedetomidine (OR = 2.40; 95% CI: 0.94–6.17; *P* = 0.062). Between-study heterogeneity was negligible (I^2^ = 0%; *P* = 0.703), and all contributing studies directionally favored higher bradycardia risk with dexmedetomidine, consistent with its known α2-agonist sympatholytic pharmacology. The large confidence interval and failure to reach statistical significance are primarily attributable to the small total number of events (24 cases across all studies), which substantially limits statistical power. The forest plot is presented in Figure 8.

#### 3.3.4 Hypotension

**Figure 9:**
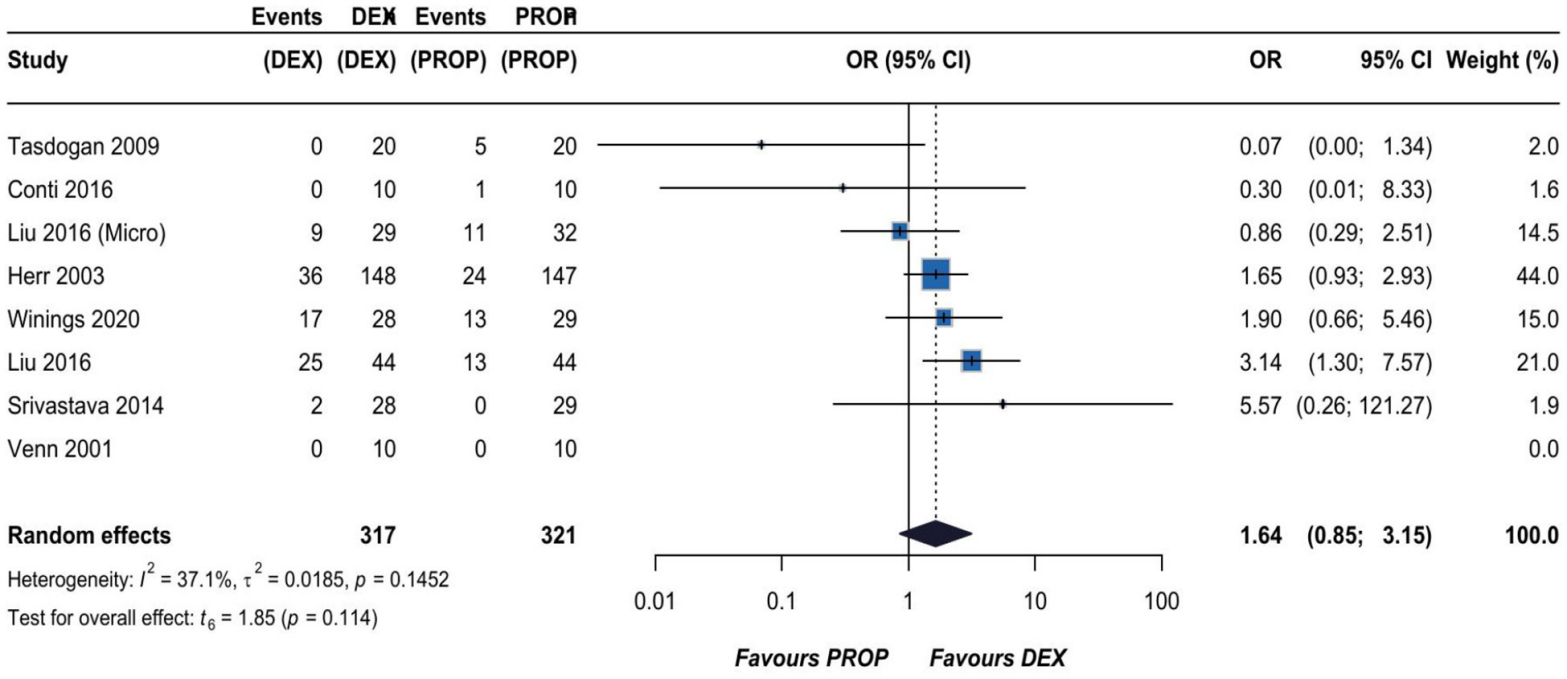
Hypotension — forest plot (dexmedetomidine vs. propofol). OR, odds ratio; CI, confidence interval.

Eight studies had hypotension data; seven (3, 18, 21–23, 26, 28) (DEX: *n* = 317; PROP: *n* = 321) were included in the pooled analysis. The pooled estimate showed a non-statistically significant trend toward higher hypotension risk with dexmedetomidine (OR = 1.64; 95% CI: 0.85–3.15; *P* = 0.114). Moderate heterogeneity was present (I^2^ = 37.1%; *P* = 0.145), partly attributable to inconsistent hypotension definitions across trials. Liu 2016-AF (23) (OR = 3.14; *P* < 0.05) and Herr (3) (weight 44.0%; OR = 1.65) were the two studies most influential in determining the direction of effect. The forest plot is presented in Figure 9. Pooled estimates for all four binary outcomes are summarized in Table 3.

**Table 3:** Summary of meta-analysis results for binary outcomes.

Outcome	Studies (k)	*N*	OR (95% CI)	*P*	I^2^ (%)	*P* heterogeneity
All-cause mortality	9	1,535	0.92 (0.76, 1.12)	0.354	0.0%	0.947
Delirium incidence	8	1,156	0.52 (0.33, 0.80)	0.010^*^	27.6%	0.208
Bradycardia	6	758	2.40 (0.94, 6.17)	0.062	0.0%	0.703
Hypotension	7	638	1.64 (0.85, 3.15)	0.114	37.1%	0.145

OR, odds ratio; CI, confidence interval. ^*^P < 0.05 (statistically significant). OR < 1 favors dexmedetomidine for mortality and delirium; OR > 1 favors propofol for bradycardia and hypotension. Mortality time-points varied across studies. Delirium tools: CAM-ICU, CAM, DSM-IV.

### 3.4 Publication bias

**Figure 10:**
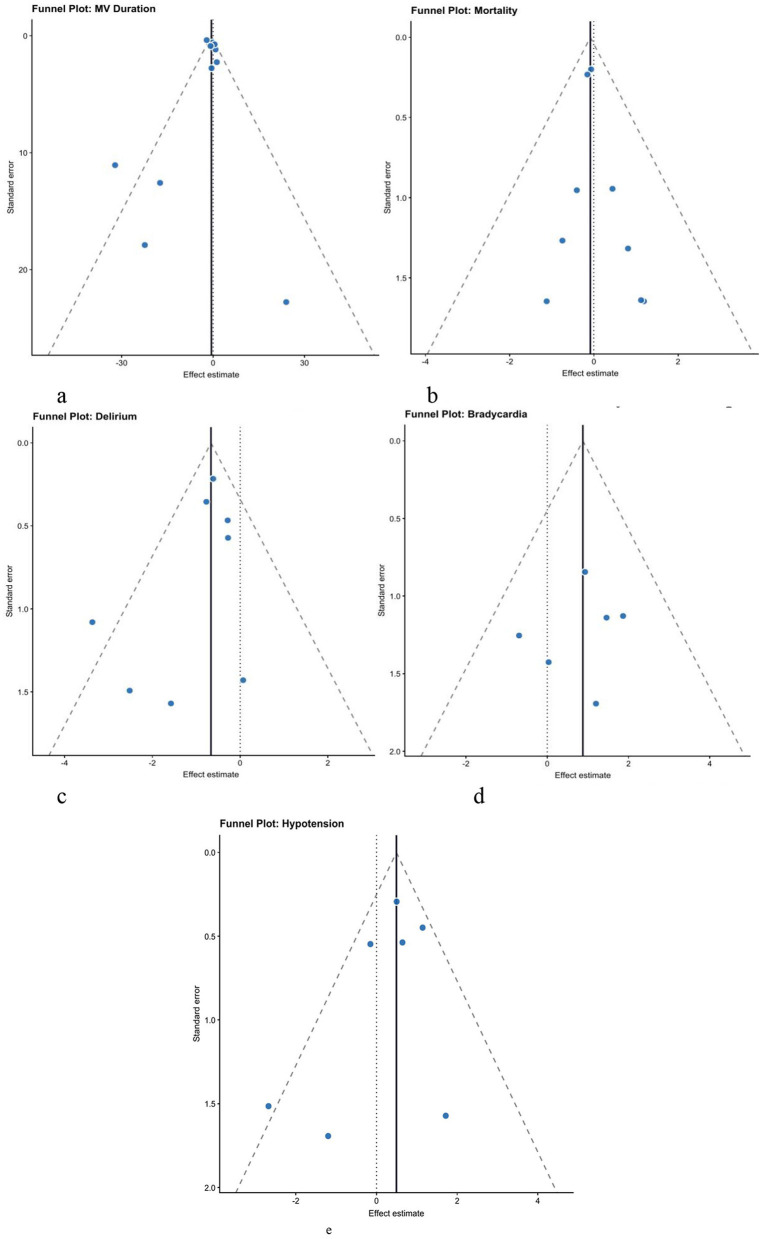
**(a)** MV duration — funnel plot. **(b)** All-cause mortality — funnel plot. **(c)** Delirium incidence — funnel plot. **(d)** Bradycardia — funnel plot. **(e)** Hypotension — funnel plot.

Funnel plots were constructed for MV duration, mortality, delirium, bradycardia, and hypotension (Figures 10a–e). The mortality funnel (k = 9; Figure 10b) showed broadly symmetric distribution, suggesting a limited impact of publication bias. The delirium funnel (k = 8; Figure 10c) displayed overall leftward displacement of effect points, consistent with a dexmedetomidine benefit; however, given only eight contributing studies, statistical power for Egger's test was insufficient, and assessment of publication bias must consider the comprehensiveness of the search strategy alongside funnel plot morphology. The MV duration funnel (Figure 10a) exhibited asymmetry at the base, reflecting the high heterogeneity of this outcome rather than simple publication bias. Funnel plots for bradycardia (Figure 10d; k = 6) and hypotension (Figure 10e; k = 7) are provided for reference only and formal publication bias testing was not performed.

### 3.5 Subgroup analyses

#### 3.5.1 MV duration by patient population type

**Figure 11:**
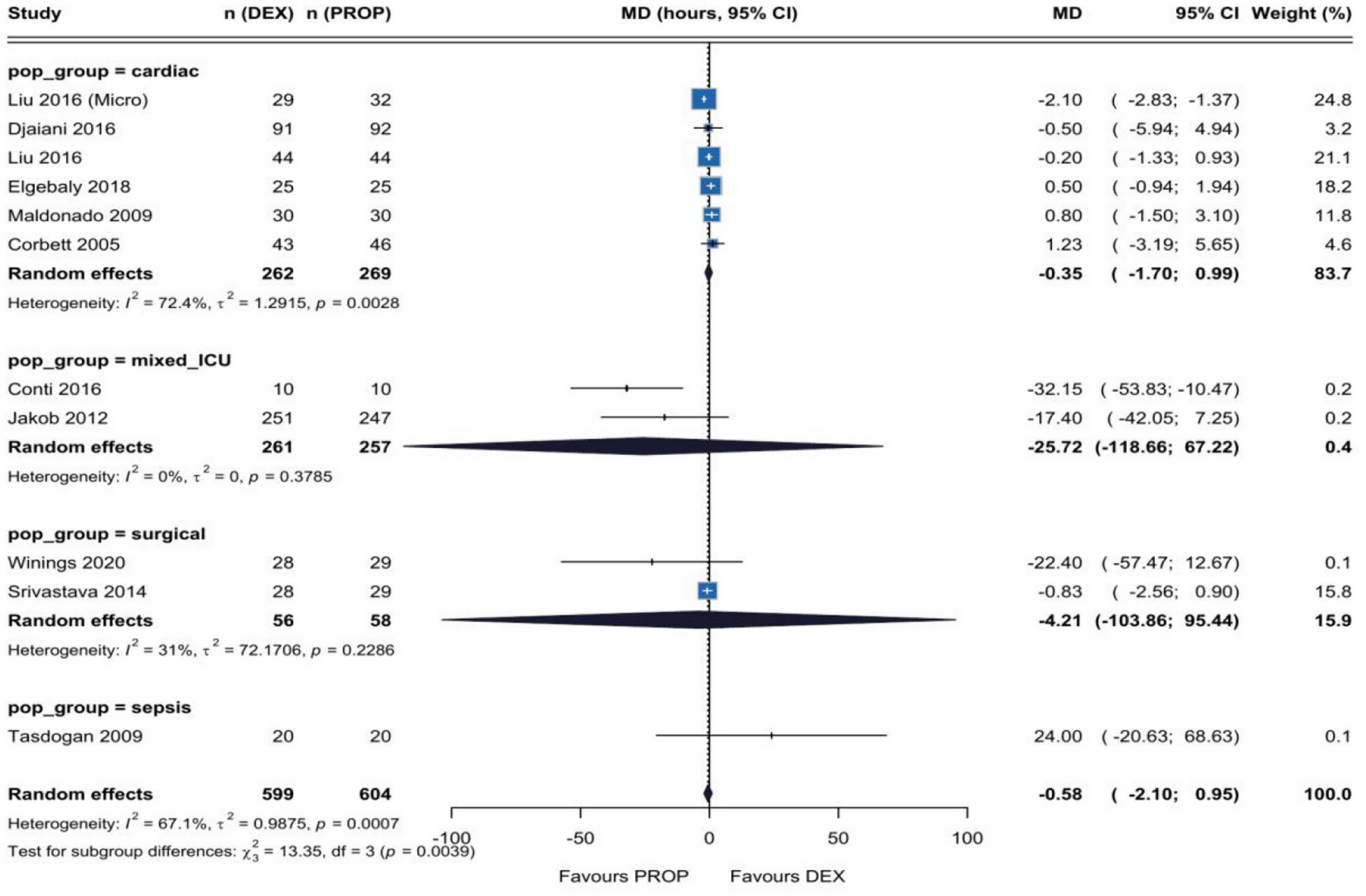
MV duration — subgroup analysis by patient population type. MD, mean difference; CI, confidence interval.

Subgroup analysis by ICU population type (Figure 11) revealed a statistically significant between-subgroup interaction (χ^2^ = 13.35; df = 3; *P* = 0.0039). The cardiac surgical subgroup (1, 19, 23–25, 28) (6 studies; *n* = 531) yielded MD = −0.35 h (95% CI: −1.70 to 0.99), without significant difference, and moderate within-group heterogeneity (I^2^ = 72.4%). The mixed ICU subgroup (4, 22) (2 studies; *n* = 518) yielded MD = −25.72 h (95% CI: −118.66 to 67.22), with an extremely wide confidence interval reflecting the markedly contrasting designs of Conti 2016 and Jakob 2012. The surgical subgroup (18, 26) (2 studies; *n* = 114) yielded MD = −4.21 h (95% CI: −103.86 to 95.44). The sepsis subgroup comprised only one study [Tasdogan (21); MD = 24.00 h; wide CI]. Subgroups containing only one study (Tasdogan 2009 in sepsis) should be interpreted as individual study results, not pooled subgroup estimates, and interaction tests involving such subgroups may be unreliable. These results suggest that ICU population type is a significant modifier of heterogeneity in MV duration, with cardiac surgical patients showing the most homogeneous and near-null response to both sedatives.

#### 3.5.2 Delirium incidence by patient population type

**Figure 12:**
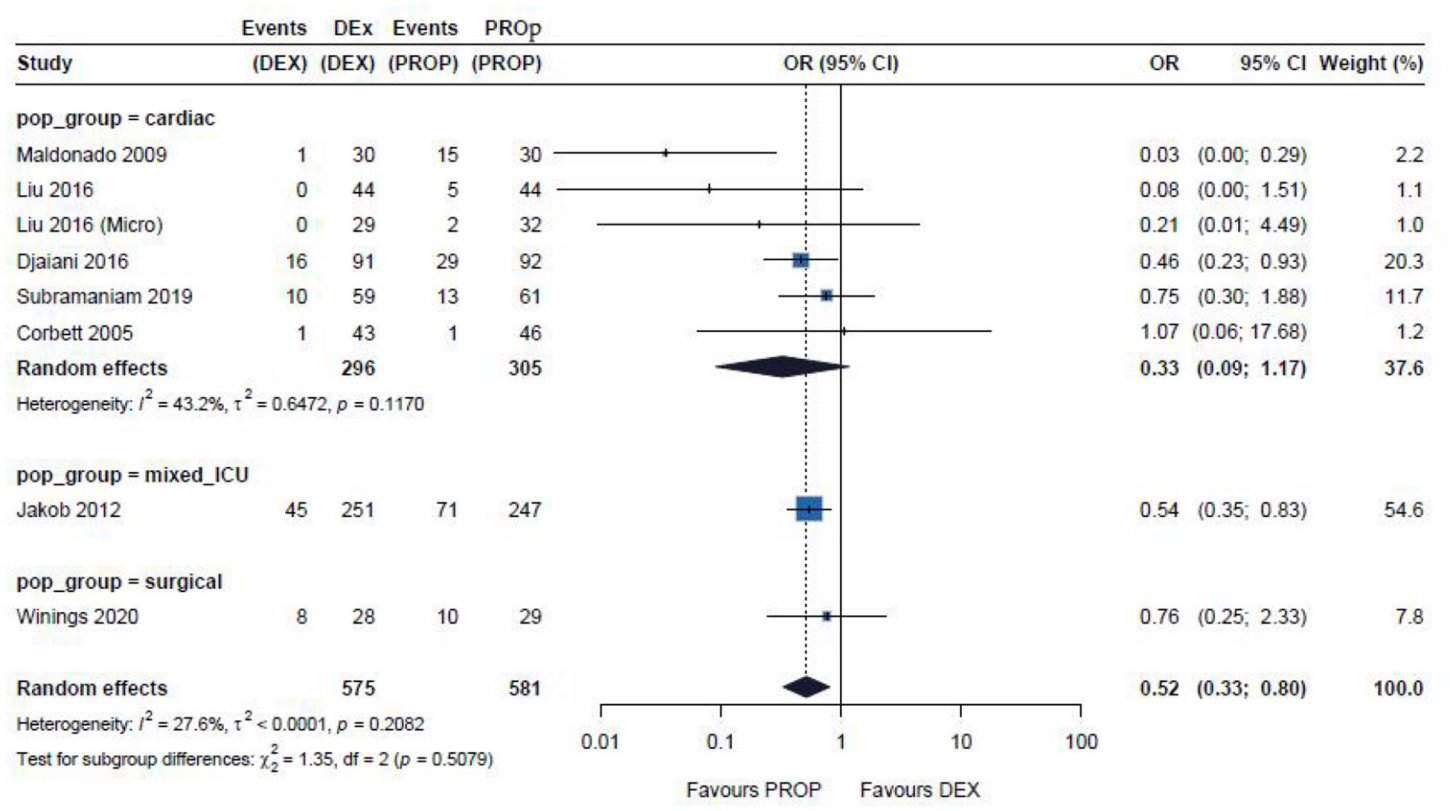
Delirium incidence — subgroup analysis by patient population type. OR, odds ratio; CI, confidence interval.

Subgroup analysis of delirium incidence by patient population type (Figure 12) did not identify a statistically significant between-subgroup interaction (χ^2^ = 1.35; df = 2; *P* = 0.508). The cardiac surgical subgroup (1, 19, 20, 23, 25, 28) (6 studies; *n* = 601) yielded OR = 0.33 (95% CI: 0.09–1.17), directionally favoring dexmedetomidine but not reaching significance owing to wide confidence intervals from predominantly small studies. The mixed ICU subgroup [Jakob (4) alone; *n* = 498] yielded OR = 0.54 (95% CI: 0.35–0.83), statistically significant. The surgical subgroup [Winings (18); *n* = 57] yielded OR = 0.76 (95% CI: 0.25–2.33). All subgroups showed consistent effect direction favoring dexmedetomidine, and the non-significant between-subgroup interaction suggests that the delirium benefit may be generalisable across ICU population types.

#### 3.5.3 MV duration by sedation duration

**Figure 13:**
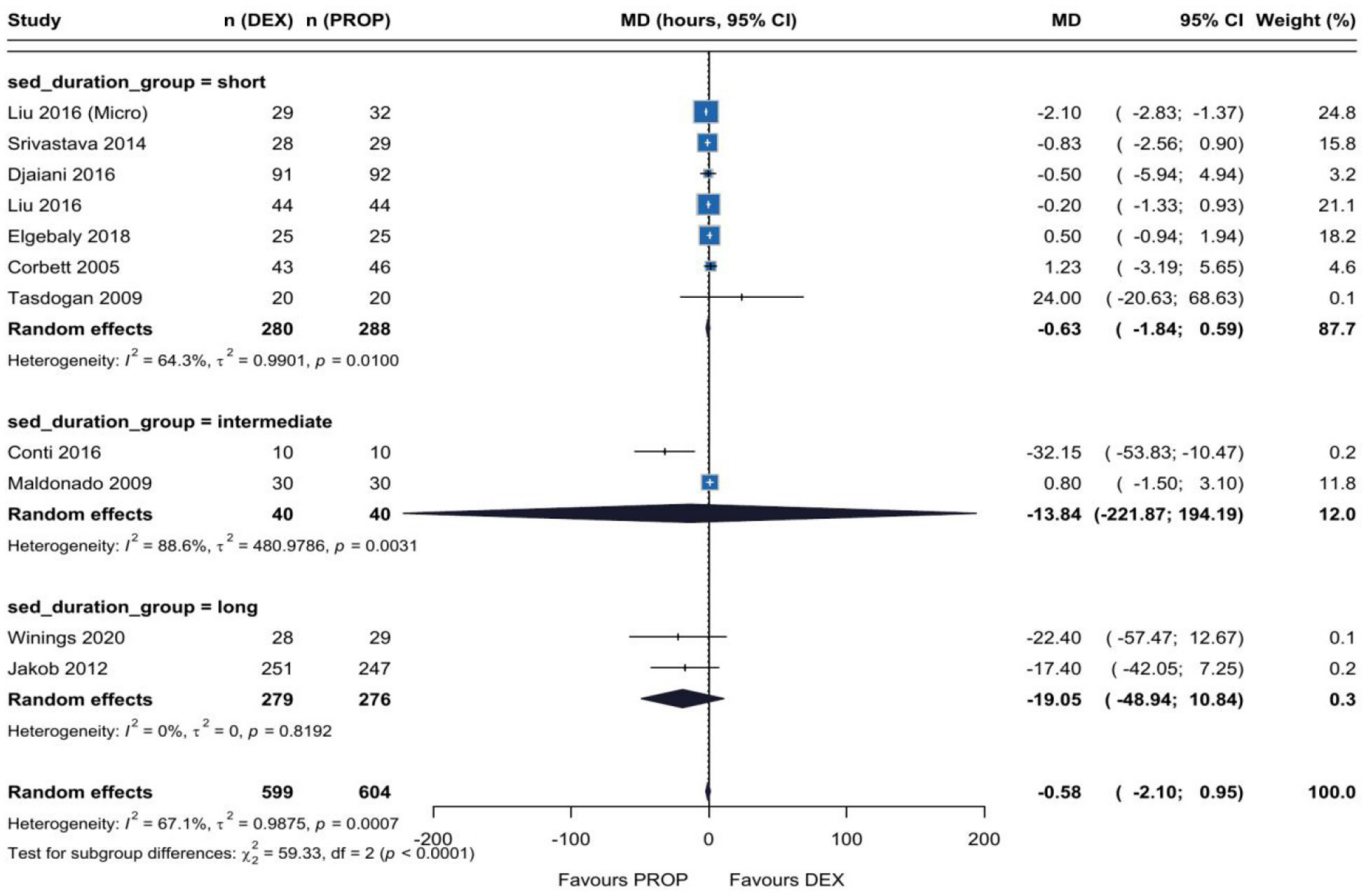
MV duration — subgroup analysis by sedation duration category. MD, mean difference; CI, confidence interval.

Subgroup analysis by sedation duration (Figure 13) identified a statistically significant between-subgroup interaction (χ^2^ = 59.33; df = 2; *P* < 0.0001). The short-duration subgroup [< 24 h; 7 studies (1, 21, 23–26, 28); *n* = 568] yielded MD = −0.63 h (95% CI: −1.84 to 0.59; I^2^ = 64.3%), without significant difference. The intermediate-duration subgroup [24–72 h; 2 studies: Conti 2016 (22) and Maldonado (19); *n* = 80] yielded MD = −13.84 h (95% CI: −221.87 to 194.19; I^2^ = 88.6%), largely driven by the extreme effect of Conti 2016 (MD = −32.15 h). The long-duration subgroup [>72 h; 2 studies: Winings (18) and Jakob (4); *n* = 555] yielded MD = −19.05 h (95% CI: −48.94 to 10.84; I^2^ = 0%), without significant difference. In summary, sedation duration is an important explanatory factor for MV duration heterogeneity, although the small number of studies within each sedation-duration subgroup limits the strength of conclusions.

### 3.6 Sensitivity analyses

#### 3.6.1 MV duration sensitivity analysis

**Figure 14:**
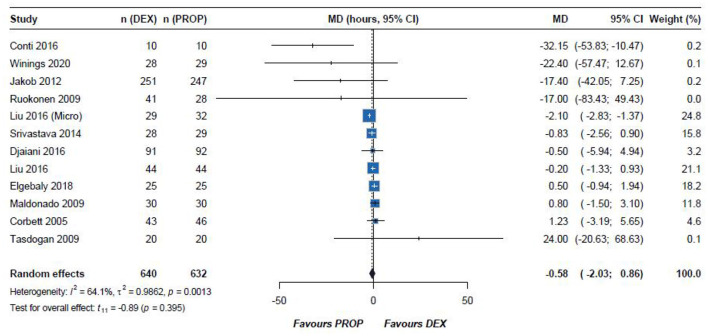
MV duration — sensitivity analysis forest plot (all 12 studies including sensitivity-analysis studies). MD, mean difference.

Of the four sensitivity-analysis studies—Ruokonen (15), Shehabi (10) (SPICE III), Kawazoe (16) (DESIRE), and Memiş (17)—only Ruokonen (15) contributed MV duration data (the remaining three either did not report MV duration directly or used non-comparable metrics). After incorporating this study, the re-pooled MV duration (12 studies; DEX: *n* = 640; PROP: *n* = 632) was MD = −0.58 h (95% CI: −2.03 to 0.86; *P* = 0.395; I^2^ = 64.1%), entirely consistent with the primary analysis conclusion. The persistence of substantial heterogeneity indicates that this variability is primarily driven by intrinsic differences in patient baseline characteristics and outcome measurement timing rather than by the exclusion criteria applied in the primary analysis (Figure 14).

#### 3.6.2 Mortality sensitivity analysis

**Figure 15:**
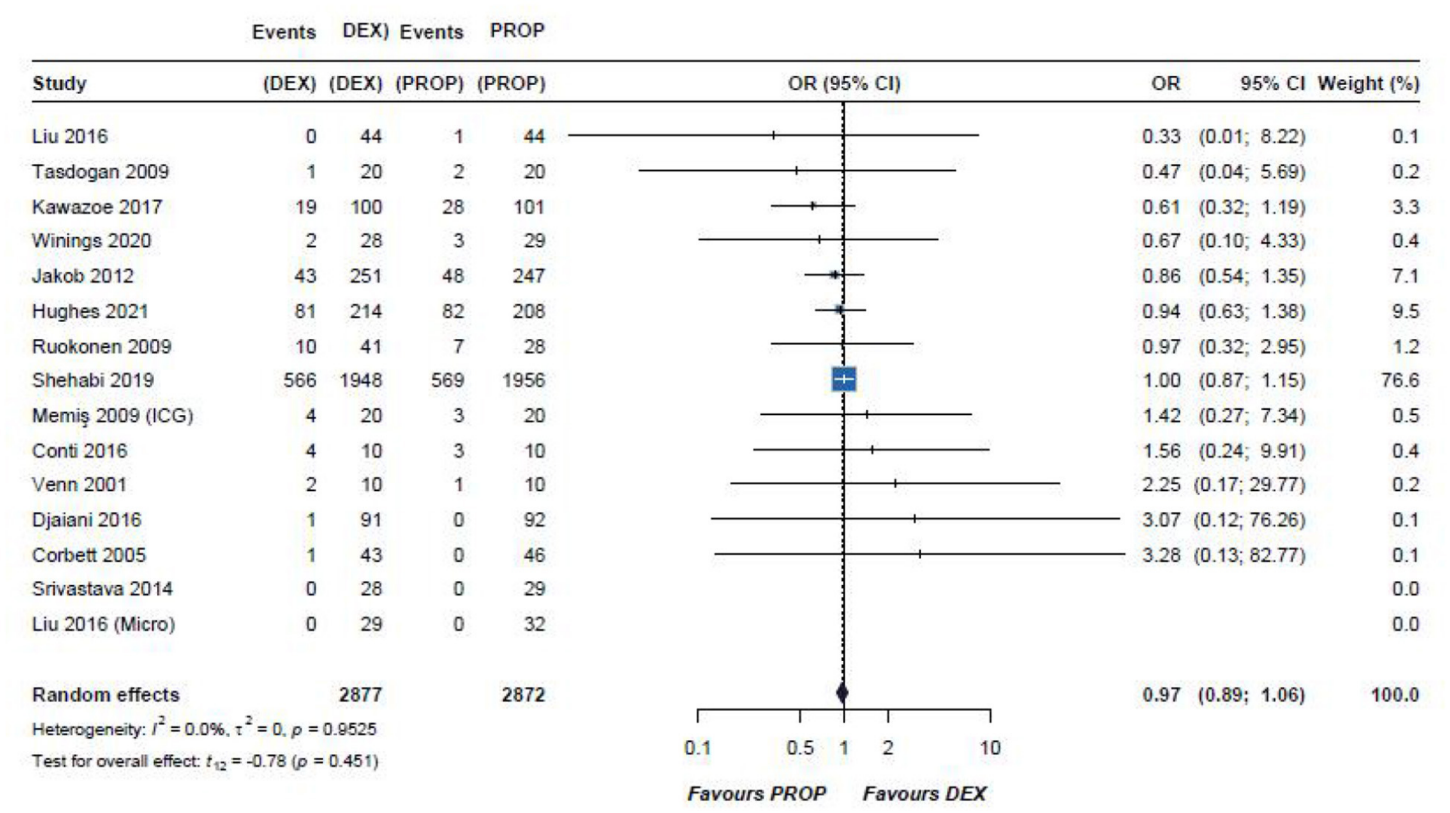
Mortality — sensitivity analysis forest plot (all 15 studies). OR, odds ratio.

After incorporating all sensitivity-analysis studies (15 studies total; DEX: *n* = 2,877; PROP: *n* = 2,872), the re-pooled mortality estimate was OR = 0.97 (95% CI: 0.89–1.06; *P* = 0.451; I^2^ = 0%), highly consistent with the primary analysis (Figure 15). The inclusion of Shehabi (10) (SPICE III) (*n* = 4,000; weight 76.6%) dramatically expanded the total sample while I^2^ remained at 0%, providing a directional consistency check, though this analysis relaxes the strict head-to-head comparison because SPICE III used a usual-care comparator arm comprising both propofol (~60%) and midazolam (~12%); accordingly, this analysis should not be interpreted as confirmation of the head-to-head estimate but rather as evidence of directional consistency. Because several trials reported low event counts, a Peto odds ratio analysis of the primary mortality data was also performed and produced a consistent null result (Peto OR = 0.92; 95% CI: 0.69–1.22; *P* = 0.568; I^2^ = 0%; Supplementary Figure S1).

### 3.7 Risk of bias assessment

Risk of bias results for the 15 primary-analysis studies are presented in Figure 2. Overall, four studies (26.7%) were rated at low risk, ten (66.7%) at some concerns, and one (6.7%) at high risk of bias, using the three canonical RoB 2.0 categories. The two highest-weighted studies—Hughes (9) (MENDS2) and Jakob (4) (PRODEX)—were rated as low risk and some concerns, respectively, providing strong methodological support for the reliability of the pooled estimates. Winings (18) was rated as high risk of bias, principally because bed-location assignment rather than true randomization was used and post-hoc statistical power was only 31.4%; however, this study's weight in the mortality analysis was only 2.3%, limiting its impact on the overall conclusion. The majority of studies (66.7%) were rated as having some concerns, primarily related to absence of allocation concealment and open-label design, limitations inherent to ICU sedation trials in which blinding of clinicians and patients to the sedative agent is operationally difficult.

### 3.8 Summary of all outcomes

**Figure 16:**
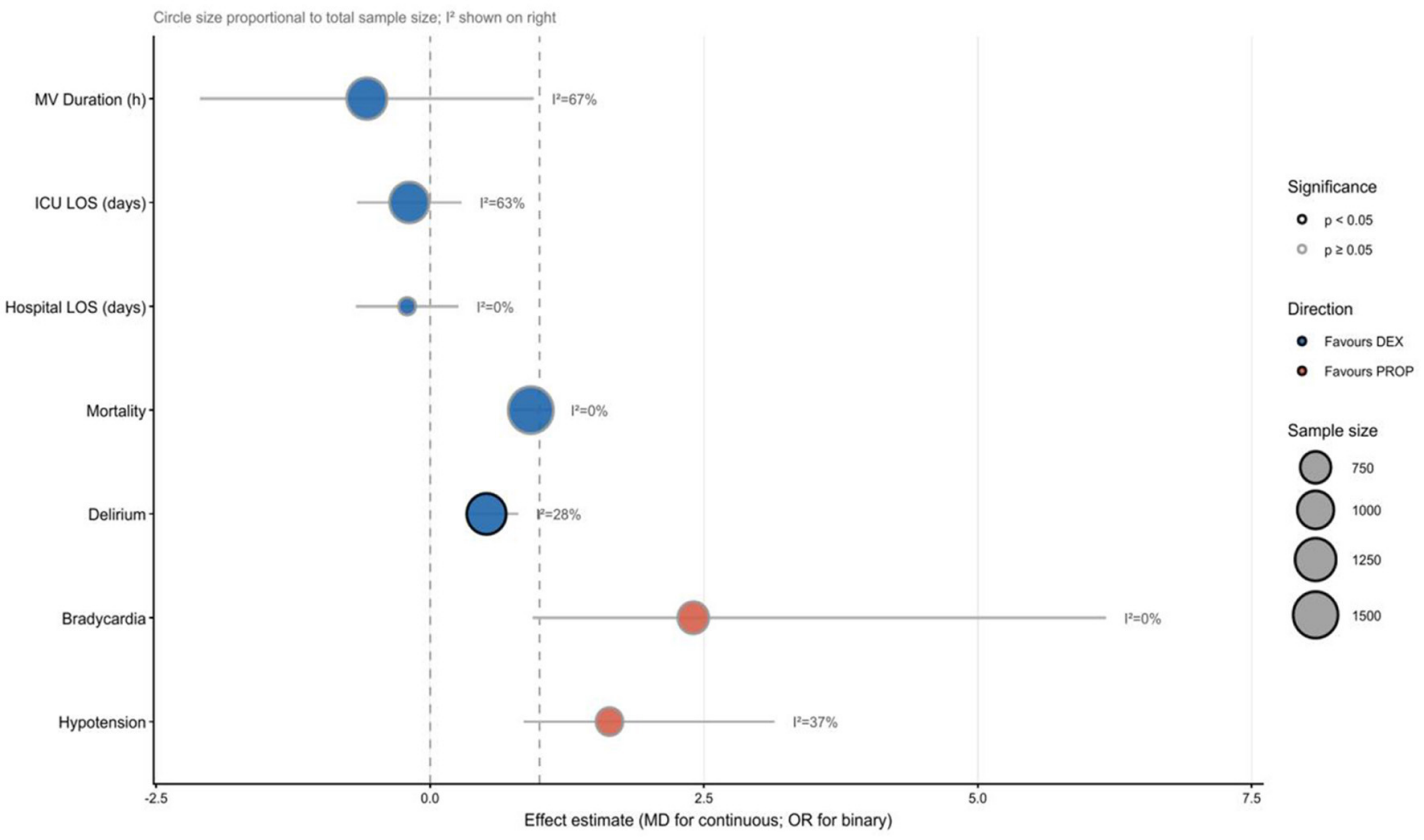
Summary bubble plot of all seven meta-analyzed outcomes. Bubble size proportional to total N analyzed. Blue, dexmedetomidine benefit; orange-red, propofol relative benefit. Dark border, statistically significant (*P* < 0.05).

Figure 16 summarizes all seven meta-analyzed outcomes in a single bubble plot. Bubble size represents total sample size for each outcome analysis; color indicates effect direction (blue: dexmedetomidine benefit; orange-red: propofol relative benefit); bubble border color distinguishes outcomes that did and did not achieve statistical significance.

Three continuous outcomes (MV duration, ICU LOS, hospital LOS) showed small effect sizes with confidence intervals crossing zero; MV duration (I^2^ = 67%) and ICU LOS (I^2^ = 63%) had substantial heterogeneity. The mortality point estimate was close to unity (OR = 0.92; I^2^ = 0%), highly consistent across studies. Among the secondary outcomes, delirium had the lowest *P*-value (OR = 0.52; shown with dark border); however, this does not survive Bonferroni correction for seven outcomes and is therefore best regarded as a hypothesis-supporting signal rather than a definitive benefit. Bradycardia (OR = 2.40; I^2^ = 0%) and hypotension (OR = 1.64; I^2^ = 37%) both indicate a relatively higher adverse event risk with dexmedetomidine, though neither achieved statistical significance. Comprehensive outcome data are summarized in Table 4.

**Table 4:** Comprehensive summary of all meta-analysis outcomes.

Outcome	Metric	*N* (total)	Effect (95% CI)	*P*	I^2^ (%)
MV duration	MD	1,203	−0.58 (−2.10, 0.95) h	0.419	67.1%
ICU LOS	MD	1,155	−0.19 (−0.67, 0.29) days	0.388	63.3%
Hospital LOS	MD	508	−0.21 (−0.68, 0.26) days	0.284	0.0%
All-cause mortality	OR	1,535	0.92 (0.76, 1.12)	0.354	0.0%
Delirium incidence	OR	1,156	0.52 (0.33, 0.80)	0.010^*^	27.6%
Bradycardia	OR	758	2.40 (0.94, 6.17)	0.062	0.0%
Hypotension	OR	638	1.64 (0.85, 3.15)	0.114	37.1%

MD, mean difference; OR, odds ratio; LOS, length of stay. ^*^P < 0.05. Negative MD or OR < 1 favors dexmedetomidine. I^2^ ≥ 50% indicates substantial heterogeneity.

## 4 Discussion

This systematic review and meta-analysis of 15 head-to-head RCTs (*N* = 1,603) synthesizes head-to-head evidence comparing dexmedetomidine with propofol sedation in adult ICU patients receiving mechanical ventilation. Both pre-specified primary outcomes showed no statistically significant difference between the two sedatives. Among secondary outcomes, dexmedetomidine was associated with a lower incidence of ICU delirium compared with propofol (OR = 0.52; 95% CI: 0.33–0.80; *P* = 0.010), however, this *P*-value (0.010) does not survive Bonferroni correction for seven outcomes, and should be interpreted as a hypothesis-supporting signal requiring dedicated confirmation. No significant differences were observed in MV duration, ICU or hospital LOS, or all-cause mortality. Dexmedetomidine was associated with numerically higher rates of bradycardia and hypotension, but neither reached statistical significance, likely owing to the small total number of adverse events across studies.

### 4.1 Comparison with existing literature

The delirium finding in the present study is consistent with the largest and most methodologically comprehensive direct DEX vs. PROP meta-analysis published to date. Heybati and colleagues (2022; British Journal of Anesthesia; 41 RCTs, *N* = 3,948) (31). similarly found that dexmedetomidine significantly reduced delirium risk in cardiac surgical patients (RR = 0.50; 95% CI: 0.29–0.87; high certainty of evidence). The MENDS2 trial (Hughes and colleagues, 2021) (9) found no significant difference between dexmedetomidine and propofol in the number of days alive without delirium or coma in septic ICU patients; however, the trial was not powered specifically for delirium incidence as a binary outcome, and differences in study design and outcome definitions may account for the discrepancy with the pooled estimate observed in the present meta-analysis. Our somewhat larger pooled effect size (OR = 0.52 vs. RR = 0.50) reflects the inclusion of non-cardiac surgical patients in our pooled analysis and the application of HKSJ CI correction.

Our null finding for MV duration (MD = −0.58 h; *P* = 0.419) contrasts with the statistically significant but clinically modest reduction reported by Heybati and colleagues in cardiac surgical patients alone (MD = −0.67 h; *P* = 0.041). This discrepancy is explained by two factors: first, our broader population inclusion introduced high heterogeneity (I^2^ = 67.1%), particularly from Conti (22) in the mixed ICU subgroup; second, the HKSJ correction produces wider confidence intervals than the standard DerSimonian-Laird approach, reducing the chance of a spuriously significant pooled estimate when k is small. The complete absence of mortality heterogeneity (I^2^ = 0%) across nine primary studies, reinforced by the sensitivity analysis including SPICE III (10) (OR = 0.97; I^2^ = 0%), provides the highest-confidence conclusion of this review: dexmedetomidine and propofol are equivalent in terms of patient survival in the ICU.

The bradycardia signal (OR = 2.40; all studies directionally consistent; I^2^ = 0%) is clinically important despite not reaching statistical significance. The zero heterogeneity confirms this is a consistent pharmacological effect rather than random variation, reflecting the well-established α2-agonist sympatholytic mechanism of dexmedetomidine. Clinicians should pre-emptively monitor heart rate and consider baseline cardiac conduction status before initiating dexmedetomidine, particularly in patients with pre-existing bradyarrhythmias or haemodynamic dependency on elevated heart rates.

### 4.2 Relevance to neurology

Although the majority of included trials enrolled post-cardiac surgical patients, delirium is a neurocognitive outcome with direct relevance to Frontiers in Neurology. ICU delirium is independently associated with long-term cognitive impairment, increased risk of subsequent dementia, and worse neurological outcomes at follow-up. The pharmacological mechanism underlying the delirium-sparing effect of dexmedetomidine—α2-adrenergic modulation of the locus coeruleus, promoting EEG patterns that resemble natural non-REM sleep rather than the cortical suppression produced by GABAergic agents—is fundamentally neurological. Two of the included studies [Srivastava (26), James (12)] specifically enrolled neurosurgical ICU patients, and larger dedicated trials in neurological and neurocritical care populations are warranted.

### 4.3 Strengths and limitations

This meta-analysis has several important strengths. First, strict inclusion criteria limited analyses to pure head-to-head DEX vs. PROP RCTs, eliminating the confounding from mixed comparators that has limited prior reviews and ensuring all effect estimates reflect the direct comparison of clinical interest. Second, use of REML τ^2^ estimation and HKSJ CI correction represents the most statistically conservative approach currently recommended for random-effects meta-analyses with small k, reducing the risk of false-positive pooled estimates. Third, pre-specified subgroup analyses by both ICU population type and sedation duration provide actionable clinical stratification. Fourth, assessment of all 15 primary studies by RoB 2.0 ensured systematic evaluation of methodological quality, and the two highest-weighted studies were both rated at low risk or some concerns, providing a robust evidential foundation.

Several limitations must be acknowledged. The primary analysis includes only 15 studies, limiting statistical power for subgroup analyses—particularly for sepsis (k = 2) and surgical (k = 2) subgroups where conclusions remain preliminary. High heterogeneity in MV duration (I^2^ = 67.1%) and ICU LOS (I^2^ = 63.3%) substantially limits the clinical interpretability of pooled estimates for these outcomes, and the sources of heterogeneity extend beyond the subgroups examined to include unmeasured factors such as concomitant opioid regimens, spontaneous breathing trial protocols, and institutional sedation cultures. Nine of 15 studies used open-label designs, introducing risk of performance and detection bias, particularly for subjective outcomes such as delirium. Inconsistent adverse event definitions across trials preclude definitive quantification of absolute haemodynamic risk. Several studies reported MV duration and ICU LOS as medians with interquartile ranges; conversion to mean ± SD using the Wan et al. method assumes approximate normality and may overestimate the mean. A sensitivity analysis restricted to studies reporting mean ± SD directly (Supplementary Figure S2) yielded broadly consistent results (k = 4; MD = 0.17 h; *P* = 0.708; I^2^ = 0%), supporting robustness. Finally, variability in delirium assessment methods (CAM-ICU, CAM, DSM-IV) and assessment frequency may have contributed to underestimation of true delirium incidence differences.

### 4.4 Clinical implications

Based on the evidence synthesized in this review, the primary clinical indication for selecting dexmedetomidine over propofol in mechanically ventilated ICU patients is the potential prevention of delirium; however, because this benefit is statistically borderline after correction for multiple comparisons, it should be weighed as a supporting consideration rather than a definitive indication. Clinicians should not anticipate clinically important reductions in MV duration or mortality from this choice. For patients in whom delirium prevention is a priority—particularly elderly post-cardiac surgical patients and those with prior cognitive impairment or established delirium risk factors—dexmedetomidine represents a pharmacologically rational choice. For patients requiring rapid sedation titration, those with significant haemodynamic instability, or those with pre-existing bradyarrhythmias or high-degree atrioventricular block, propofol may remain the more controllable and haemodynamically safer option.

## 5 Conclusions

In mechanically ventilated adult ICU patients, neither pre-specified primary outcome (MV duration, ICU LOS) differed between dexmedetomidine and propofol. Dexmedetomidine may reduce ICU delirium (OR = 0.52; *P* = 0.010), but this secondary finding is borderline after adjustment for multiple comparisons. No evidence of a mortality difference between agents is supported by negligible heterogeneity across both primary and sensitivity analyses. The consistent bradycardia signal across all contributing studies warrants clinical vigilance and pre-emptive monitoring. These findings suggest that when delirium prevention is prioritized, dexmedetomidine warrants consideration, while propofol may remain preferable in haemodynamically unstable patients or those with pre-existing bradyarrhythmias. Larger, adequately powered RCTs stratified by ICU subpopulation—particularly in septic and neurocritical care patients—are needed to further refine individualized sedation decision-making.

## Data Availability

The original contributions presented in the study are included in the article/Supplementary material, further inquiries can be directed to the corresponding authors.
