# Supplementary material for: Dexmedetomidine vs. propofol for sedation in mechanically ventilated ICU patients: a systematic review and meta-analysis of randomized controlled trials
Source: Front Neurol. 2026 Jul 22;17:1875240. doi: 10.3389/fneur.2026.1875240 (PMC13437391; doi:10.3389/fneur.2026.1875240)
Supplement: Supplementary file 1 [file Supplementary_file_1.pdf]

Supplementary Materials

Dexmedetomidine vs. Propofol for Sedation in Mechanically Ventilated Adults:

A Systematic Review and Meta-Analysis of Randomized Controlled Trials

Contents

Table S1. Study–Outcome Inclusion Matrix

Figure S1. Sensitivity Analysis of Mortality — Peto Odds Ratio

Figure S2. Sensitivity Analysis of MV Duration — Studies Reporting Mean ± SD Only

Figure S3. Sensitivity Analysis of ICU LOS — Including Elgebaly 2018

Table S1. Study–outcome inclusion matrix. Each row represents a study included in the systematic review; columns represent the seven clinical outcomes. "✓" indicates the study contributed data to the pooled analysis for that outcome (including studies that reported data but were excluded from quantitative synthesis due to zero events in both arms). "—" indicates the study did not report the outcome.

Study	MV Duration	ICU LOS	Hospital LOS	Mortality	Delirium	Bradycardia	Hypotension
Corbett 2005	✓	✓	—	✓	✓	—	—
Maldonado 2009	✓	✓	✓	—	✓	—	—
Conti 2016	✓	✓	—	✓	—	✓	✓
Liu 2016	✓	✓	✓	✓	✓	✓	✓
Elgebaly 2018	✓	—	—	—	—	—	—
Djaiani 2016	✓	✓	✓	✓	✓	—	—
Hughes 2021	—	—	—	✓	—	—	—
Venn 2001	—	—	—	✓	—	✓	✓
Srivastava 2014	✓	—	—	✓	—	✓	✓
Liu 2016 (Micro)	✓	—	—	✓	✓	✓	✓
Jakob 2012	✓	✓	—	✓	✓	—	—
Herr 2003	—	—	—	—	—	✓	✓
Subramaniam 2019	—	✓	✓	—	✓	✓	—
Winings 2020	✓	✓	✓	✓	✓	✓	✓

Study	MV Duration	ICU LOS	Hospital LOS	Mortality	Delirium	Bradycardia	Hypotension
Tasdogan 2009	✓	✓	—	✓	—	✓	✓

Abbreviations: MV, mechanical ventilation; ICU, intensive care unit; LOS, length of stay.

Notes: Elgebaly 2018 reported ICU LOS but was excluded from the primary pooled analysis (k = 9) because of a disproportionately short observation window; a sensitivity analysis including this study is presented in Figure S3. For mortality, Srivastava 2014 and Liu 2016 (Micro) reported zero events in both arms and were therefore excluded from the pooled risk-ratio analysis. For bradycardia, three of nine reporting studies had zero events in both arms and were excluded from the pooled analysis. For hypotension, one of eight reporting studies had zero events and was excluded.

Figure S1. Sensitivity analysis of mortality using the Peto odds ratio method.

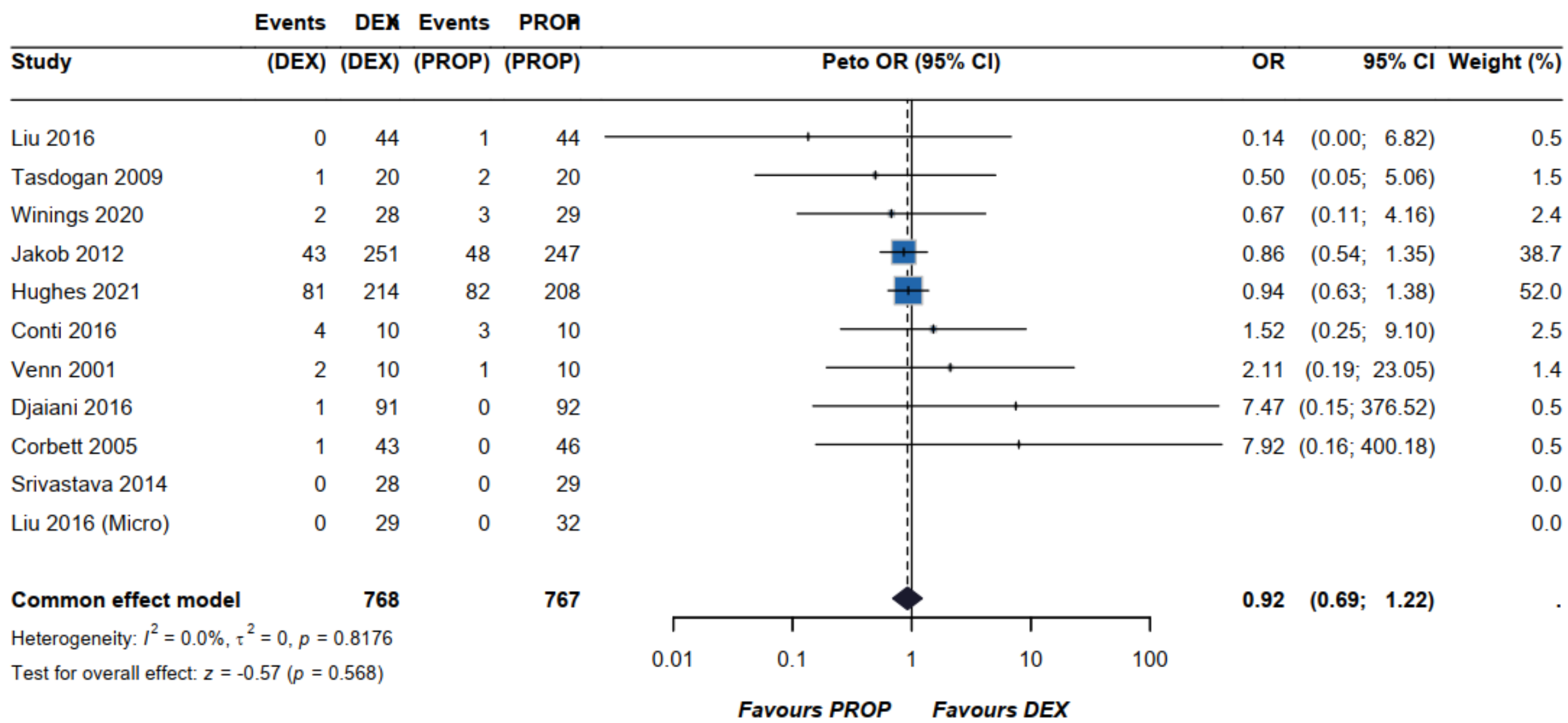

Forest plot of mortality comparing dexmedetomidine with propofol, analyzed using the Peto fixed-effect method to account for low event rates across trials. The pooled Peto OR was 0.92 (95% CI: 0.69–1.22; P = 0.568; $I^2 = 0\%$), consistent with the primary inverse-variance analysis showing no significant difference in mortality between groups. File: FigS1_Sensitivity_Mortality_Peto.pdf

Figure S2. Sensitivity analysis of MV duration restricted to studies directly reporting mean ± SD.

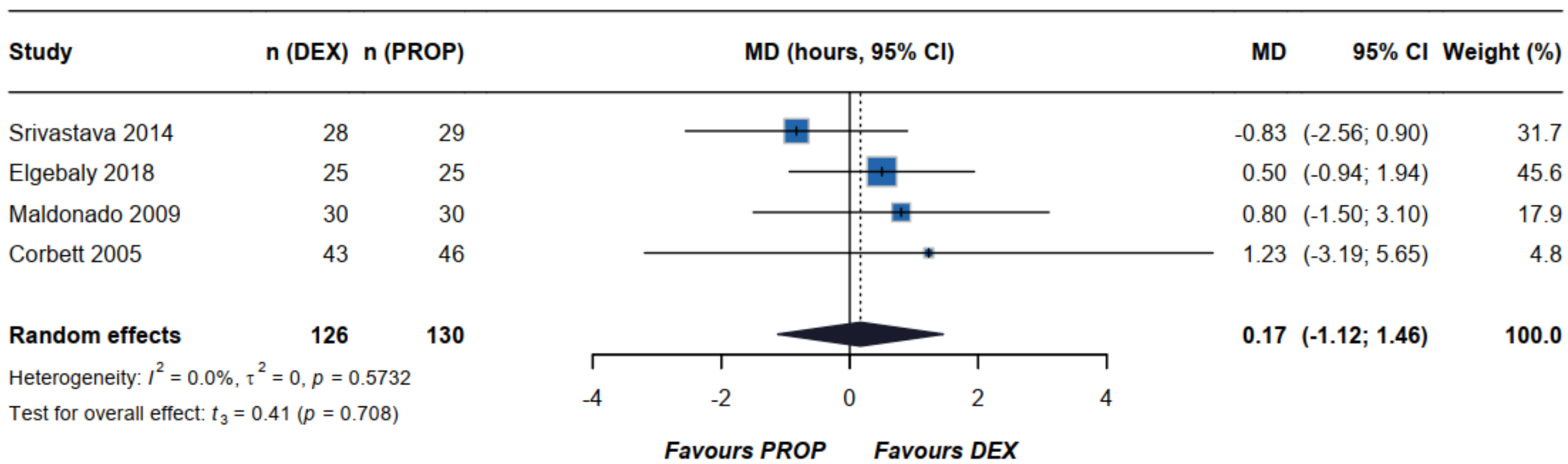

Forest plot of mechanical ventilation duration including only the four studies that directly reported mean and standard deviation (excluding studies where median/IQR was converted). The pooled MD was 0.17 hours (95% CI not significant; P = 0.708; I^2 = 0%), indicating that conversion of non-parametric summary statistics did not materially influence the primary analysis result. File: FigS2_Sensitivity_MV_MeanSD_only.pdf

Figure S3. Sensitivity analysis of ICU LOS including Elgebaly 2018.

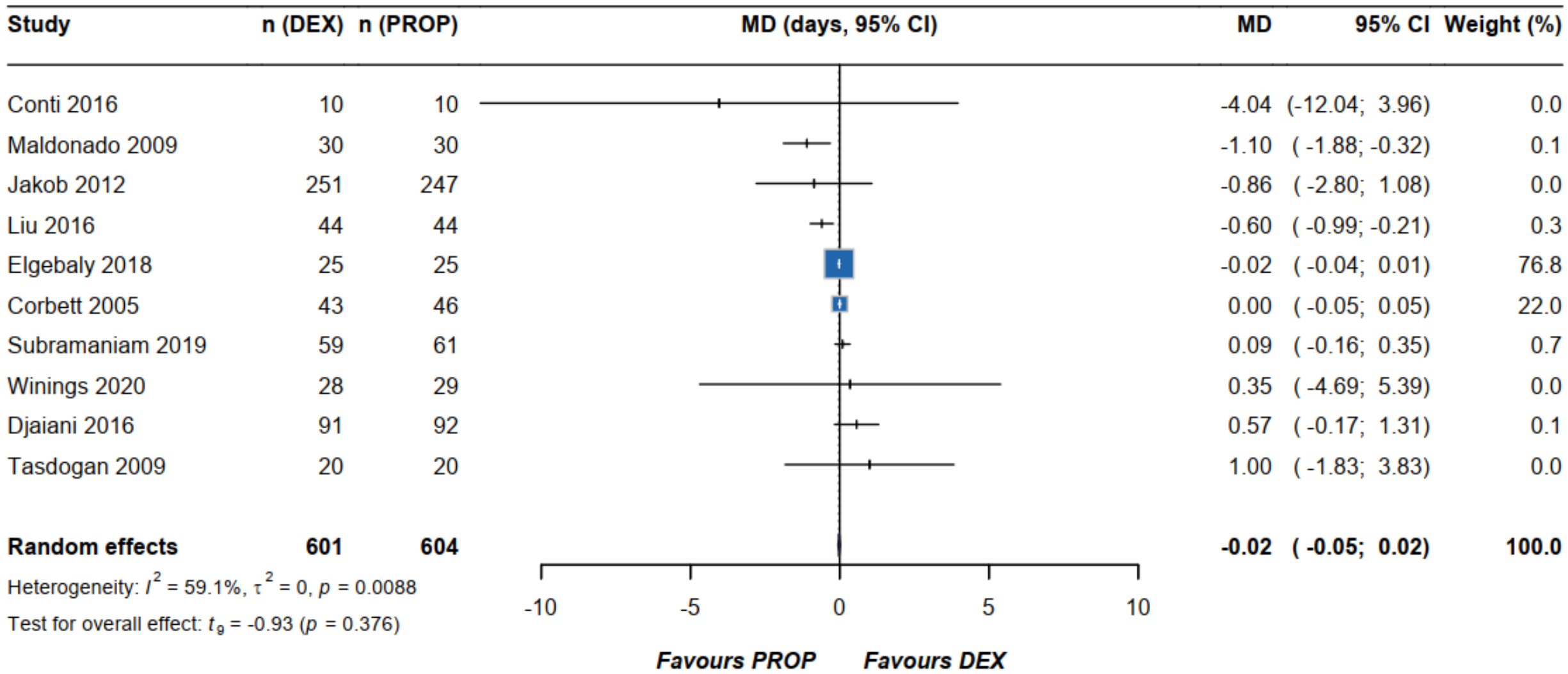

Forest plot of ICU length of stay with Elgebaly 2018 reintroduced into the pooled analysis (k = 10). Elgebaly 2018 carried a dominant weight of 76.8% due to its very narrow confidence interval. The pooled MD was −0.02 days (95% CI: −0.05 to 0.02), confirming that inclusion of this study did not alter the overall finding of no significant difference in ICU LOS between dexmedetomidine and propofol. File: FigS3_Sensitivity_ICU_LOS_with_Elgebaly.pdf
